# Dr. Charles E. Simmons

**Published:** 1917-06

**Authors:** 


**Dr. Charles E. Simmons,** Gottingen 1862, M. D. P. & S. 1864, died at his home in N. Y., May 3, aged 76. He was formerly Commissioner of Charities and Corrections.

